# SOCIAL SUPPORT AND SOCIAL RESOURCES PREDICTING LIFE SATISFACTION AMONG CENTENARIANS AND OCTOGENARIANS

**DOI:** 10.1093/geroni/igz038.620

**Published:** 2019-11-08

**Authors:** Rotem Arieli, Peter Martin, Leonard Poon

**Affiliations:** 1 Iowa State University, Ames, Iowa, United States; 2 University of Georgia, Athens, Georgia, United States

## Abstract

Although much research has assessed the relationship between social support and life satisfaction for older adults, there is little information on how social support predicts life satisfaction over and above social resources among very old people. The purpose of this research was to determine pathways from demographic variables, social resources, and social support to life satisfaction. Data from 208 cognitively-intact centenarians and octogenarians of the Georgia Centenarian Study (GCS) were analyzed using multiple regression analyses to evaluate pathways from social resources via social support to life satisfaction. Three different models were analyzed in the GCS sample: one with a combined group of octogenarians and centenarians, one with only octogenarians, and one with only centenarians. Path models included: demographic variables (gender, ethnicity, residential type, and age in years) to social resources to social provisions to life satisfaction. Results in the combined older adult group showed that residence type significantly predicted social resources, β = -.26, p < .01, social resources significantly predicted social provisions, β =.15, p < .05, and social provisions significantly predicted life satisfaction, β =.15, p < .05. Results in the centenarian sample showed that both residence type and age significantly predicted social resources, β =-.19, p < .05, and β = -.17, p = .05, respectively, and social resources significantly predicted social provisions, β = .18, p = .05. Overall, results indicate the uniqueness of the centenarian population and their paths to high life satisfaction through social resources and support.

